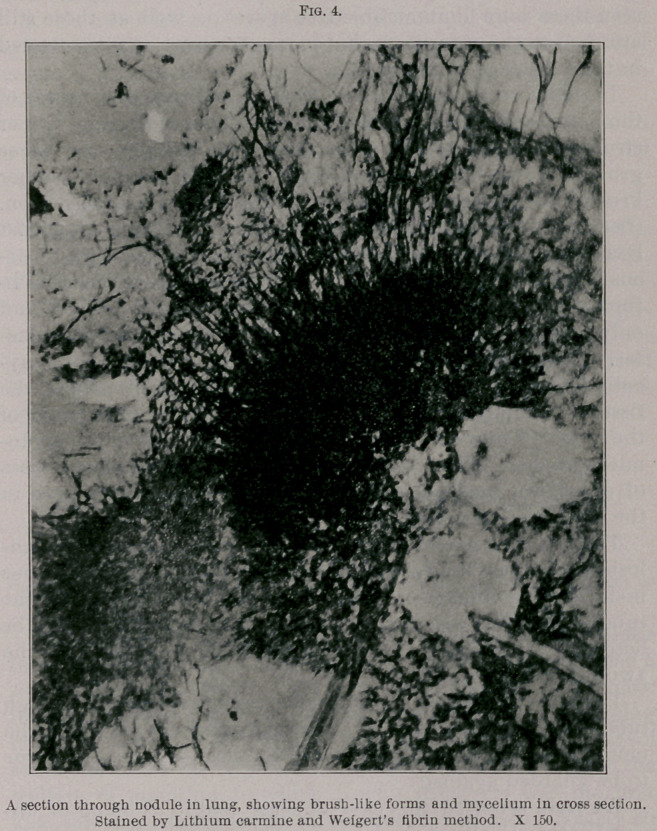# A Case of Pneumonomycosis Due to the Aspergillus Fumigatus1Read before the Pathological Society of Philadelphia May, 24, 1900. (The cuts used in this article are from the University Medical Magazine.)

**Published:** 1900-08

**Authors:** Leonard Pearson, Mazyck P. Ravenel

**Affiliations:** Professor of Theory and Practice of Veterinary Medicine and Dean of the Faculty of Veterinary Medicine, University of Pennsylvania; State Veterinarian of Pennsylvania; Bacteriologist of the State Live-Stock Sanitary Board of Pennsylvania; Lecturer and Demonstrator of Bacteriology, Veterinary Department, University of Pennsylvania


### THE JOURNAL
OF
### COMPARATIVE MEDICINE AND VETERINARY ARCHIVES.

VOL. XXI. *AUGUST, 1900.* No. 8.

### A CASE OF PNEUMONOMYCOSIS DUE TO THE ASPERGILLUS FUMIGATUS.[1]

BY LEONARD PEARSON, B.S., V.M.D.,
PROFESSOR OF THEORY AND PRACTICE OF VETERINARY MEDICINE AND DEAN OF THE FACULTY OF VETERINARY MEDICINE, UNIVERSITY OF PENNSYLVANIA; STATE VETERINARIAN OF PENNSYLVANIA,

AND

MAZYCK P. RAVENEL, M.D.,
BACTERIOLOGIST OF THE STATE LIVE-STOCK SANITARY BOARD OF PENNSYLVANIA; LECTURER AND DEMONSTRATOR OF BACTERIOLOGY, VETERINARY DEPARTMENT, UNIVERSITY OF PENNSYLVANIA.

(*From the Laboratory of the State Live-stock Sanitary Board of Pennsylvania.*)

*Historical.* The history of mould mycoses is of great interest. In the year 1815 Mayer observed in the bronchi, air-sacs, and lungs of a jay a mould, the first recorded case of parasitism by a fungus of this class.

From this time a number of instances were observed in birds, but it was not until 1857 that Rivoltá published the first case recorded of mycotic infection in a mammal, the subject being a horse, which had a tumor of the pharynx, in the pus of which mould mycelium was demonstrated. In the meantime, however, in 1842, Hughes Bennett had found a fungus in the sputum, cavities, and nodules of a phthisical patient, the first case of mycotic infection observed in man. Others made similar observations, though Sluyter, in 1847, was the first to clearly demonstrate an aspergillus as the fungus in a case of pneumonomycosis. In 1856 Virchow published the reports of four cases of bronchial and pulmonary invasion by the aspergillus in patients who succumbed to other affections, the invasion being regarded as secondary, an opinion generally held up to

[1] Read before the Pathological Society of Philadelphia May, 24, 1900. (The cuts used in this article are from the University Medical Magazine.)

the time of the Berlin congress in 1890, when Dieulafoy, Chantemesse, and Widal reported their observations on the disease, as seen in the pigeon-feeders of Paris, together with their experimental studies, and for the first time took the ground that the affection was a primary one, and not secondary or terminal. In 1897, Rénon published his "Étude sur l' Aspergillose chez les Animaux et chez l'Homme," the most complete and masterly presentation of the subject which had appeared in any country. Rénon has made a careful study of the disease as seen in man and animals spontaneously, and has also produced it experimentally a great many times. From the vast amount of evidence thus obtained he maintains successfully the position first affirmed by Dieulafoy, Chantemesse, and Widal, namely, the occurrence of a primary pneumo-aspergillosis. In England cases of the primary infection have been reported by Boyce and Arkel and Hinds.

Rénon gives an interesting description of aspergillosis as a trade-disease among the pigeon-feeders and hair-sorters of Paris. The men who perform the operation of "gavage" in pigeons take into their mouths a mixture of millet- and vetch-seeds in water, which they force into the mouths of the birds. The hair-sorters use rye-flour in considerable quantities to absorb the grease, so often in hair, to enable them to disentangle the knots. The air in the work-rooms is irrespirable. Birds kept in these rooms die in two or three weeks, coughing and becoming much emaciated; dogs survive some three months, and cats alone are able to resist infection.

Quite recently, 1900, our knowledge has been extended by an important monograph, entitled "Pneumonomykosis Aspergillina," by Dr. F. Saxer, of Jena, Germany. He reports four new cases of aspergillar mycosis observed by himself in man, and gives the details of a large amount of experimental work, in addition to which there is a complete bibliography and review of published cases. It is the most notable addition to the literature of the affection which has appeared since the work of Rénon.

*General considerations.* Pulmonary aspergillosis is an uncommon disease, and practically all of the exact knowledge we have of it is of recent date. While infection by other mould-fungi has been reported, it is difficult to explain just why the aspergillus fumigatus should be the offending organism in almost all cases. The high temperature at which it thrives, and the wide extent of its distribution in nature no doubt have something to do with it, but cannot account entirely for the fact.

The mode of infection is through the respiratory tract. Only a small number of the spores inspired are able to reach the alveoli, the great majority being arrested on the way, and thrown out in the tracheal and bronchial secretions. Having reached the alveoli, Hildebrandt had shown that they penetrate the epithelial lining without difficulty, and are taken up by the "staubzellen." Both animals and man appear to possess a great degree of immunity to intestinal infection. Grawitz has never observed it; Lucet has seen it only once, and Rénon has produced it only a few times experimentally.

The aspergillus forms no toxin, either intra- or extra-cellular, and its pathogenic power is due entirely to a traumatism exercised by the masses of mycelium, which leads to a necrosis of the cells, and a leucocytic reaction which diminishes the functions of the organs, the final result being an enfeebled condition of the animal and a lessened resistance to hurtful influences. When fruit hyphæ can form, the myriads of spores given off by them may be carried to other parts of the organ, and in this way the foci rapidly multiply, and, as in the case under consideration, practically the entire organ becomes invaded. The opinion held by some authors that in the mould mycoses there is "no fructification or actual multiplication" of the infecting agent, and that the "number of the disease foci corresponds exactly with the number of spores introduced," is erroneous as to the spontaneous disease as well as to the experimental form. In the latter, fruit formation is exceptional, but has been observed by Rénon, and, as in the spontaneous disease, takes place only where there is full communication with the air. It has been observed only in the lung. It is extremely rare for aspergillosis to pass from one animal to another, and infection can take place only by spores and not by mycelium.

In experimental aspergillosis, especially when produced by the injection of a small number of spores, and somewhat slow in its evolution, the nodules bear a very close resemblance to those of tuberculosis, as they are seen in the kidneys, lungs, liver, bone, etc. Rénon considers the affection a pseudo-tuberculosis, which he would designate as "aspergillar tuberculosis," while the nodules are "aspergillar tubercles." Certainly the clinical features of the affection in many cases justify the name. Clinically, in man as well as in animals, the disease presents two distinct types, while there are intermediate forms which partake of the character of both. In one form, the symptoms are almost exactly those of tuberculosis; in the second, emphysema and bronchitis are marked.

In cases where the course of the affection is chronic, the fungus may or may not be found in the tissues. When it has disappeared, a chronic interstitial pneumonia may remain which eventually causes death. In lung-tissue still containing fungus, we find dilated bronchioles, often deprived of their epithelium, leading into pneumonic areas, in which are nodules or pseudotubercules made up of mycelium, sometimes so arranged as to resemble closely actinomycosis. There is much phagocytic reaction in these pneumonic areas, indicating an active resistance to the invasion. In cases where the symptoms have been those of emphysema and dyspnœa, patches of consolidation breaking down into cavities are found, with well-marked compensatory emphysema.

Microscopically the walls of the small bronchi are thickened, and both the alveolar walls and cavities contain mycelium. In some areas the tissues are so disorganized as to be unrecognizable, and there is breaking down with the formation of microscopic cavities. The mycelium is in intimate relation with the lung-tissue, and, since it is accompanied with much phagocytic reaction, the invasion seems to be the cause of the lesions.

In old cases in animals we sometimes find foci which have become caseous or even calcareous, usually about the size of a pea, containing both pus and mycelium. Almost always the nodules alternate with areas of pneumonic consolidation and emphysema. An interesting feature is the absence of fetor in cases of gangrene of the lung associated with the aspergillus, the fungus probably inhibiting the growth of putrefactive bacteria. The formation of the "odorless gangrenous cavities of Virchow" is ascribed by Saxer to the action of the aspergillus.

Pneumonomycosis aspergillina is a rare disease among domestic animals, and has not heretofore been described in the United States in a mammal. Cadéac (*Pathologie Interne des Animaux Domestiques*, Paris, 1897), cites five reports of this disease among horses and seven among cattle. Schneidemühl

(*Lehrbuch der Vergleichenden Pathologie und Therapie*, Leipzig, 1898), states that mycotic pneumonia is not rare among animals and that it is especially common among birds. However, cases are not cited to support the statement in regard to the frequency of this condition. He states that mycotic pneumonia is more common among horses and cattle than among sheep and dogs, and that fungi are frequently accidental in the lungs. Friedberger and Fröhner (*Lehrbuch der Speciellen Pathologie und Therapie der Haustheire*, Stuttgart, 1896), quote a report from Pech, dating from the year 1875, in which it is recorded that seven horses kept in one stable and fed on mouldy, musty cut-straw developed mycotic pneumonia at the same time. Ostertag (*Handbuch der Fleischbeschau*, Stuttgart, 1899), calls attention to the resemblance between some cases of aspergillar mycosis of the lungs and tuberculosis, and, in some few instances, there is said to be a certain resemblance to the macroscopic lesions of lung-plague. The small hepatized areas in pneumonomycosis do, indeed, bear some resemblance to the lesions produced by a recent invasion of tubercle bacilli into a restricted area, but are not enough like the sharply circumscribed tubercles that one usually sees to occasion an error in diagnosis.

The resemblance to lung-plague in the present case was even more superficial. As will be seen by reference to the notes on the necropsy, the interlobular connective-tissue was emphysematous, thus causing what was seen on the surface of the lungs as yellowish bands separating the somewhat hyperæmic lobules of the lungs. This caused a distinctly "marbled" appearance, but the marbled appearance was not due to the lesions produced by the fungus; it was due entirely to the emphysema in the interlobular connective-tissue and the hyperæmia of the lobules. It was only upon a closer examination that one could detect the deep-red and comparatively small areas caused by the fungus.

The case reported by Roeckl, in 1884, is quoted by Kitt (*Lehrbuch der Pathologisch-Anatimischen Diagnostik*, Stuttgart, 1895), and is cited here to show that sometimes there may be a closer resemblance to lung-plague. In Roeckl's case the lungs of a cow presented extensive hepatization, to which was added fibrinous pleurisy. There were hemorrhages in the lung, lymph-stasis, and extensive distention of the interlobular connective-tissue, with inflammatory infiltration; thus giving the "marbled" appearance. In addition, there were in part of the lobes numerous, isolated, sharply circumscribed nodules of hemp-seed size, and on the bronchial mucous membrane isolated ulcers 4 to 8 millimetrcs across, coated with a friable mass of tissue débris. Each nodule was found to contain at its centre an aspergillus colony, with the mycelial threads radiating out into the surrounding tissue. The hepatization was due to the proximity of numerous nodules and accompanying changes in the parenchyma, consisting in capillary ectasis, hemorrhage, and infiltration of leucocytes.

*Cultivation and description of the mould.* The aspergillus fumigatus is readily cultivated artificially. It grows on almost any of the ordinary culture-media used in bacteriology, but the reaction should be acid, as it grows poorly in alkaline media. The well-known Raulin's fluid is perhaps the best material for culture, especially where the aspergillus must be isolated from mixed growths, as in the examination of sputum, etc. Sabouraud's solution of maltose, prepared according to the following formula, also gives good results: Maltose, 3 to 70 grammes; peptone, 0.75 grammes; distilled water, 100 c.c. To this may be added gelatin or agar to harden it, the latter being preferable, as the aspergillus grows best and forms fruit best at 37° to 39° C. For ordinary use no media give better results than potato, with or without glycerin, and a paste made by rubbing up crumbs of stale bread in water.

Growth is more rapid, however, in Raulin's fluid than in any other medium, mycelium appearing in from five to twelve hours, and spores forming in twelve to fifteen hours. The growth is first a velvety white, soon becoming a delicate bluish-green, which grows darker, and on Raulin's fluid changes after some days to a dark brown. Cultures on potato retain their green color for a long time, while those on bread-paste become brown in time. Cultures retain their vitality for many months unimpaired, and will give growth even after three or four years. Spores do not form below 20° C., and like the mycelium require free access to oxygen for their best development. They measure $2.5\mu$ to $3\mu$ in diameter. In nature the spores are widely distributed, but seem to be especially abundant in grain and vegetable matter. They have considerable powers of resistance to heat and to chemical agents. They are killed by moist heat at 60° C. in five and a half hours, and by bichloride of mercury, 1 to 1000, in fifteen minutes.

The aspergillus fumigatus is differentiated from other species by its color in cultures, the high temperature at which it grows, the size of its spores, and by its pathogenic power. In practice the aspergillus glaucus is the one most likely to be confounded with it, but it may be recognized by its ability to grow at low temperatures, its delicate green color, the large size of its spores—$9\mu$ to $15\mu$ in diameter—and its lack of pathogenic power.

*Case.* The animal under consideration was a Jersey cow, six years old. She was bred and raised on a farm near Philadelphia, and was never off of this farm until sent to the Veterinary Hospital a short time before she died. The herd of which this cow was a member numbers about eighty animals. The milking-cows are kept in the basement of a large stone barn. The cow-stable is lighted on three sides, but not in such a way as to allow the rays of the sun to reach more than a small fraction of it. The cows are fed from an artificial-stone floor, and are confined in stanchions with no partition to separate them from one another. The hay is thrown down into the stable from a loft above. The floor is frequently strewn with land-plaster, and the atmosphere of the stable is often loaded with dust.

This cow was in bad condition for six months; she was not thrifty, her coat was rough, the skin tight, the nutritive condition bad. During the last two months emaciation was more rapid, and the animal coughed considerably, especially after eating. The cough was dry and harsh. The appetite continued, but the flow of milk diminished to about one-fourth of the previous volume. Suspecting tuberculosis, she was tested with tuberculin, but gave no reaction. She was treated with tonics, but did not improve; on the contrary, her condition became worse, and about two weeks after the test an obstinate diarrhœa developed that could not be controlled by the ordinary farm-treatment, and the owner concluding that the cow was worthless decided that he would destroy her. In order that the animal might be observed more closely a request was made that it should be sent to the Veterinary Hospital of the University of Pennsylvania. This was done. Upon arrival at the hospital it was found that the cow would not eat, and she was weak and depressed; respirations labored, and from forty to sixty per minute; pulse rapid. Percussion of the chest-walls gave a sound that, if anything, was clearer and louder than the normal percussion sound. Upon auscultation it was found that the vesicular and bronchial murmurs were considerably increased in intensity and accompanied here and there by sibilant râles. The cow coughed violently at times. Six days after she came to the hospital the breathing became more rapid and difficult, and the pulse very much accelerated. The animal did not eat, grew weak rapidly, and died four days later, or ten days after admission to the hospital.

*Autopsy.* The animal was much emaciated. The mucous membrane of the small intestine was catarrhal and showed a small amount of erosion. All the organs were normal except the

FIG. 1.

A mass of fruit heads and free spores from a scraping of a nodule. Glycerin preparation unstained. X 400.

lungs, one of which was removed to the laboratory for minute examination. The most striking feature on external examination was the extreme amount of emphysema. The lobules were separated from each other as much as 3 to 5 millimetres, and even at some distance from the borders light could be seen through these crevices on holding the lung up before the eyes. On the surface the subpleural connective-tissue was distended in large blebs. Upon palpation the lung crackled, and numerous hard nodules could be felt. On section a curious picture was presented, numerous dark-red nodules being seen, the surrounding lung appearing normal in color. In each lung there were from fifty to sixty of these nodules, from 5 to 12 millimetres in diameter, most of them dark red, and closely resembling partially organized blood-clots. However, on crushing a por-

FIG. 2.

The same as number 1, but more highly magnified. X 500.

tion in glycerin between two slides and examining under the microscope, they were found to be made up almost entirely of a felted network of mycelial threads. Between these large nodules were numberless smaller areas of the same color, 1 to 2 millimetres in diameter, not perceptible to the touch as nodules, but which were of the same character, and were, no doubt, foci of recent origin. These were seen especially well in portions of the lung which were preserved by Pick's method, the slight bleaching of the tissues bringing them into relief. On opening some of the interlobular emphysematous spaces small whitish mouldy-looking patches were noticed on nodules which bordered the cavity. Scrapings from these patches were made up entirely of perfect fruit hyphæ with myriads of spores. (See Figs. 1 and 2.) The diagnosis of a mould mycosis was in this way made at once and confirmed by cultures and examination of sections. Cultures were made on glycerinated potato, bouillon, and plain agar, by opening a nodule with sterile instruments and tearing out a small portion of the centre, which was transferred to the culture-tubes and placed in the incubator at 39° C. Abundant growth was obtained on the potato by the end of thirty-six hours, white at first, and soon changing to yellowish-green, then to dark green. The growth in the bouillon and agar was slow. Plates and flasks of bread-paste were made, and these, with potato, were employed for all subsequent cultures. The formation of the fruit-hyphæ was studied, and the spores measured a number of times, being from $2.5\mu$ to $3.5\mu$ in diameter. By these means the culture was identified as the aspergillus fumigatus.

Our experiments on animals were limited to the inoculation of one rabbit, in the aural vein of which one-half cubic centimetre of a suspension of the spores was injected. The animal died in forty-four hours, and from the liver and kidneys cultures were recovered. All of the organs were examined in sections, but only in the same two was mycelium detected.

*Microscopic examination.* The nodules were hardened and embedded, some in celloidin, and some in paraffin. Various staining methods were tried, hæmatoxylin and eosin, carbol-thionine, Gram, and lithium carmine with Weigert's fibrin-stain. Good results were obtained with all, but the carmine and Weigert gave the most beautiful picture, and by this method the fungus was most perfectly demonstrated, the spores and mycelium taking on a deep purple color. The histological picture was studied mainly in sections stained by hæmatoxylin and eosin. The bronchial epithelium was normal in places, but for the most part the columnar cells have been replaced by a sort of membrane, which appears to be made up almost entirely of a felt-work of mycelial threads. From this membrane hyphæ grow out into the lumen of the bronchus, and here, owing, no doubt, to the supply of air, fruithyphæ arise, with perfect sterigmata and spores. There is no cellular nor other exudate and very little débris. The under surface of this membrane is of looser texture, and contains some cellular infiltration made up of round-cells, leucocytes, proliferated connective-cells and red blood-corpuscles. The adjacent structures are closely filled with a cellular infiltration of the same description, with a quantity of mycelium, this extending to the neighboring alveoli, which under a low power (× 80) appear to have preserved their outline; but with greater amplification (× 625) are seen to have lost all their normal structure, showing clumps of homogenous, irregular masses, which stain faintly with eosin, and are probably of connective-tissue origin.

In these areas the mycelium has followed the alveolar wall as a trellis, the tissue seeming to oppose no obstacle to its advance. Within the alveoli is a finely granular débris, with some coarser particles, probably the remains of cells. In sections stained with carbol-thionine large numbers of mast-cells are seen in the alveolar walls. Bordering these degenerated areas are alveoli which have retained their normal structure and are filled with a network of fibrin holding in its meshes a few cells—leucocytes and septal cells. In other parts of the sections are areas resembling those just described, but in which all anatomical landmarks have been destroyed, so that it is impossible to tell whether or not the spaces seen are bronchi.

Some sections show a widespread interstitial and alveolar hemorrhage, the blood showing a considerable increase in the number of leucocytes. The capillaries are congested, and areas of œdema with thickening of the alveolar walls are not uncommon.

There is peri-bronchitis and arteritis, while in some sections arterial thrombosis is seen, the thrombus being penetrated by mycelium, though no fruit is found. Areas are also found in which the alveoli are filled with a cellular exudate producing consolidation and thickening of the alveolar walls.

Emphysema, both interstitial and vesicular, is marked, and often extreme. Around the borders of the interstitial cavities is a distinct zone made up of red blood-cells, leucocytes, and a homogeneous material, which is yellowish in fresh as well as in stained sections. The areas contain very little mycelium. All sections show a small amount of anthracosis. The appearance of sections varies in different nodules, and in the same nodule as they are taken further and further away from the centre. In general the fungus is thickest at the centre, so thick in many instances that the lung-tissue is hidden entirely, and grows less as we go outward. The tissue changes

FIG. 3.

Section through nodule in lung, showing actinomycotic form of mould. Stained by Lithium carmine and Weigert's fibrin method. X 50.

noted, formation of fibrin, etc., take place in a zone beyond the greatest growth of the fungus. In other nodules the fungus is evenly distributed throughout, following the alveolar walls. In these the tissue changes are slight. At times the fungus grows in dense brush-like clusters, closely resembling actinomycosis under a low magnification. (See Fig. 3.) This form is considered to show a marked reaction and resistance on the part of the animal, and a lowered vitality in the fungus.

FIG. 4.

A section through nodule in lung, showing brush-like forms and mycelium in cross section. Stained by Lithium carmine and Weigert's fibrin method. X 150.

When found it indicates that the aspergillosis is a primary and not a secondary or terminal affection. No giant cells were found in any section.

Fruit formation was not observed in the substance of the tissues at any time. It was observed most frequently in bronchi, which were for the most part denuded of their epithelium, and next in emphysematous cavities, where it could be detected in clusters by the naked eye. Fruit was also found in sections, in spaces the nature of which it was impossible to determine accurately. Wherever the formation of fruit was seen there were innumerable free spores as well as those still attached to the sterigmata, but in no case were spores detected in the substance of the tissues.

In many sections, especially those from near the centre of the nodules, the mass of mycelium was so dense that the proper structure of the tissues was obscured. Besides the dense growths resembling actinomycosis, already described, other brush-like clusters, not unlike them, were frequently seen. (See Fig. 4.) These differ from the former in being somewhat less compact and in the fact that from their periphery numerous hyphæ run out into the surrounding tissues, whereas in the actinomycoctic form the masses are sharply defined, and only here and there a few threads grow out beyond the cluster. Their appearance suggests that they may be actinomycotic forms which have finally overcome the resistance of the tissues. Emphysema is less marked in the neighborhood of the latter. These differences are well brought out in the photomicrographs. When a cluster is obliquely cut we have a mass of closely-set cut-ends of mycelium in cross section as seen in the left lower corner of Fig. 4.

The cow was examined carefully for the lesions of tuberculosis, and in one lung some four or five caseous and calcareous nodules 1 centimetre in diameter were found in which the tubercle bacillus was demonstrated, but no mycelium could be detected. In no part of the lung was there coexistence of the two infections.

The fact that the animal did not react to tuberculin, though tuberculosis was present, is noteworthy and suggests that the mould infection may interfere with the test—a source of error to be guarded against.

We have reported this case at some length as being of unusual interest. We have been unable to find any record of a similar one ever having been observed in America, the literature on the subject in this country being limited to two cases in birds, and one case reported by Osler to this society in 1887, in which mycelium was found in the sputum of a woman suffering from bronchial asthma.

We consider the case one of primary aspergillosis for the following reasons:

1. The aspergillar nodules were by far the most extensive lesions found, and amply sufficient to account for the symptoms and death.

2. No other lesions were found which could have caused the symptoms, the tuberculous nodules being few in number and confined to a small portion of one lung. They were also evidently not progressive.

3. The actinomycotic forms of the aspergillus were found in a number of the nodules, indicating strong resistance on the part of the tissues.

In conclusion, we wish to express our thanks to Dr. C. Y. White, of the Pepper Clinical Laboratory, for assistance in the histological examination of the nodules, and to Mr. Wm. H. Walmsley for his beautiful photomicrographs.

We have used freely the article on "Pulmonary Aspergillosis" by Rolleston, in *Allbutt's System of Medicine*, the monograph of Saxer, and especially the most admirable work of Rénon, to the authors of which we make grateful acknowledgments.

## Figures and Tables

**Fig. 1. f1:**
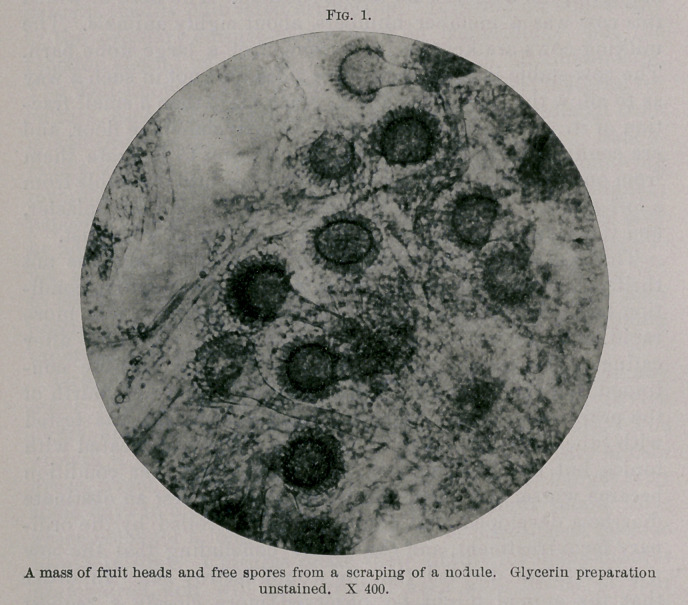


**Fig. 2. f2:**
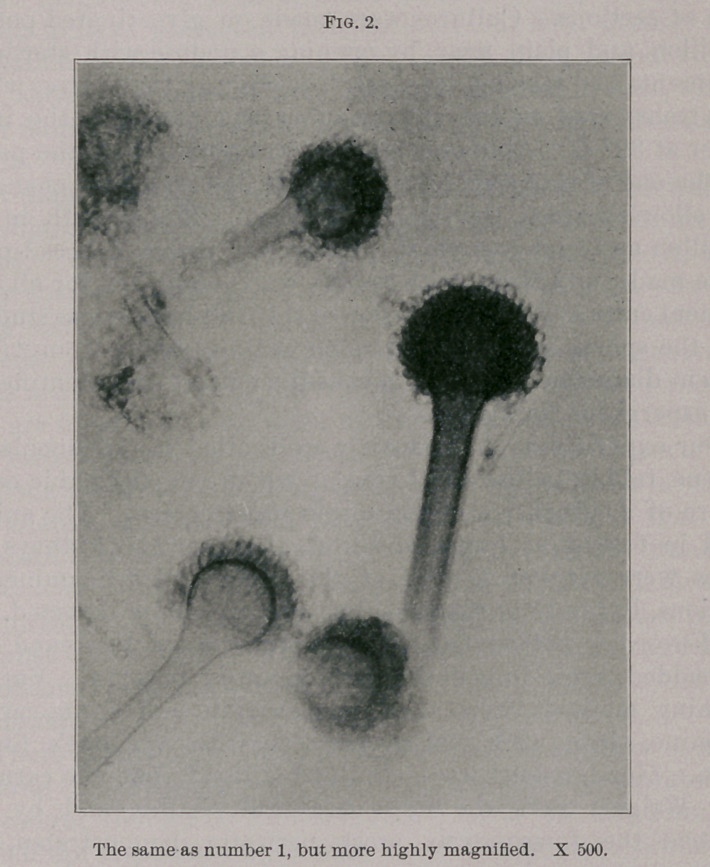


**Fig. 3. f3:**
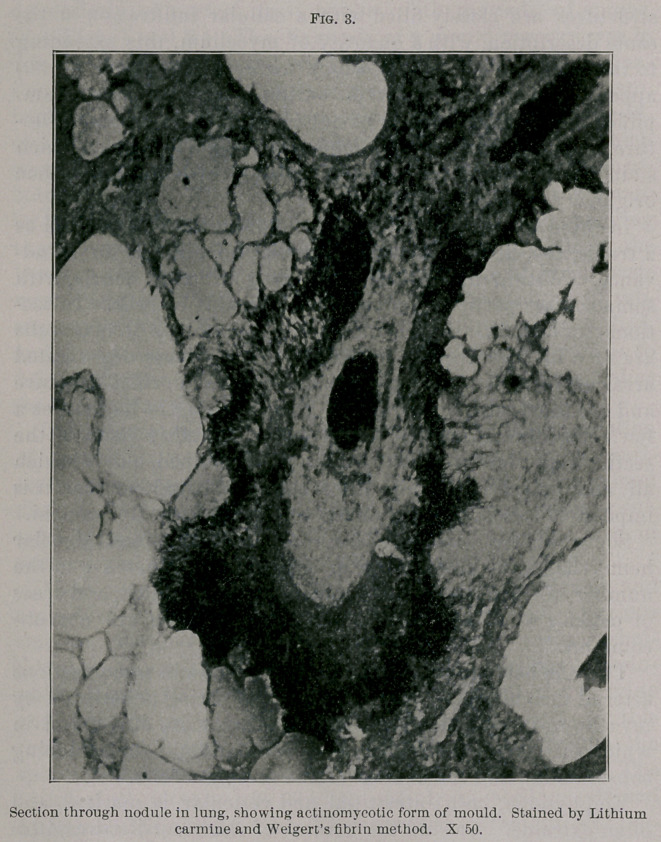


**Fig. 4. f4:**